# Mississippi State Medical Society

**Published:** 1882-10-20

**Authors:** 


### *TRANSACTIONS OF THE MISSISSIPPI STATE MEDICAL SOCIETY.*

The Society met at Oxford, on April 5th, 1882. The volume before us contains 170 pages plainly and neatly gotten out.

The next place of meeting is appointed at Meridian on the 1st Wednesday in April, 1883.

The following are the contents of the volume, among which are some very creditable papers:

President's address, by Dr. B. F. Wood: Alcohol.

New Remedies, by Dr. B. F. Kittrell; Senecio Lobatus, by Dr. D. L. Phares; Retained Placenta, by Dr. T. R. Trotter; Scarlet Fever, by Dr. F. W. Daney; Traumatic Tetanus, by Dr. H. A. Grant; Abortive treatment of Puerperal Convulsions, by Dr. T. H. Gordon; Infantile Convulsions, by Dr. J. P. Moore; Puerperal Eclampsia, successfully treated with Veratrum viride, with cases, by Dr. N. L. Guice; Case of Perforation of the Illium by Worms, by Dr. T. J. Crofford; Three cases of Embryotomy, by Dr. J. D. Talbert; Spinal Curvature, by Dr. Chesley Daniel; Surgical cases, by Dr. W. R. Blailock.

REPORT ON THE SURGERY OF MISSISSIPPI—By Dr. M. S. Craft. Cases reported by Dr. T. J. Crofford, of Coffeeville; Dr. R. C. Cunningham, of Verona; Dr. Geo. C. Phillips, of Lexington; Dr. J. M. Taylor, of Corinth; Dr. D. C. Montgomery, of Greenville; Dr. R. E. Jones, of Crystal Springs; Dr. E. L. McGehee, of Woodville; Dr. J. E. Halbert, of Leota Landing; Dr. J. M. Green, of Aberdeen; Dr. S. V. D. Hill, of Macon; Dr. R. E. Edwards, of Edwards; Dr. F. W. Daney, of Holly Springs; Dr. W. R. Blailock, of Carthage; Dr. W. B. Sanford, of Corinth; Dr. C. R. Henderson, of Deasonville; Dr. R. M. Young, of Corinth; Dr. J. H. Blanks, of Meridian; Dr. M. S. Craft, of Jackson.

Obituary—Dr. W. P. Finley.

The following are the officers for the present year:

*President*—Wirt Johnston, M.D., Jackson.
*Vice-Presidents*—1st. J. M. Griffin, M.D., Aberdeen.
2d. J. E. Halbert, M.D., Leota Landing.
3d. J. T. Chandler, M.D., Oxford.
4th. E. L. McGehee, M.D., Woodville.
*Recording Secretary.*—T. W. Fullilove, M.D., Vaiden.
*Corresponding Secretary.*—M. S. Craft, M.D., Jackson.
*Treasurer.*—Robert Kells, M.D., Jackson.
*Orator.*—G. W. Trimble, M.D., Grenada.
*Alternate Orator.*—J. B. Sanford, M.D., Corinth.

